# Increased expression of cyclin E is associated with an increased resistance to doxorubicin in rat fibroblasts

**DOI:** 10.1038/sj.bjc.6600970

**Published:** 2003-06-10

**Authors:** A Sgambato, A Camerini, G Pani, R Cangiano, B Faraglia, G Bianchino, B De Bari, T Galeotti, A Cittadini

**Affiliations:** 1 Istituto di Patologia Generale, Centro di Ricerche Oncologiche ‘Giovanni XXIII’, Catholic University, Largo Francesco Vito 1, 00168 Rome, Italy

**Keywords:** cyclin E, rat fibroblasts, doxorubicin, drug resistance

## Abstract

Cell cycle progression in eukaryotic cells is regulated by a family of cyclin-dependent kinases (CDKs). Cyclin E is a regulatory subunit of CDK2 and drives cells from G1 to S phase. Increased expression of cyclin E is a frequent event in human malignancies and has been associated with poor prognosis in various cancers. In this study, we evaluated the effects of cyclin E-overexpression on the sensitivity of rat fibroblasts to anticancer drugs. Cyclin E-overexpressing cells were less sensitive to doxorubicin-induced inhibition of cell growth but not to other antineoplastic drugs, such as paclitaxel, vincristine, etoposide and methotrexate. Cyclin E-overexpressing fibroblasts also displayed a reduction in ROS levels and a significantly lower increase following doxorubicin treatment compared with vector control cells. The expression of manganese superoxide dismutase (MnSOD) and its activity were increased (about 1.3-fold) in cyclin E-overexpressing derivatives compared with control cells. These results suggest that cyclin E overexpression might reduce tumour cells sensitivity to doxorubicin by affecting the expression of MnSOD and that determination of cyclin E expression levels might help to select patients to be treated with an anthracycline-based antineoplastic therapy.

In mammalian cells, progression through the different phases of the cell cycle is regulated by the activity of a series of cyclin-dependent kinases (CDKs) that are activated by cyclins, their regulatory subunits. Cyclin E is a G1 cyclin expressed near the G1 to S transition and drives entry into the S phase by binding to and activating CDK2. Accumulation of cyclin E in late G1 is achieved by periodic transcription coupled with regulated ubiquitin-mediated proteolysis of the protein (Clurman *et al*, 1996). The proper timing and amplitude of cyclin E expression are important since their alterations cause deregulation of cell growth (Ohtsubo *et al*, 1995; Sgambato *et al*, 1997). Moreover, transgenic mice overexpressing cyclin E develop breast cancers (Bortner and Rosenberg, 1997) and increased levels of cyclin E have been reported in a variety of human malignancies, (Donnellan and Chetty, 1999; Sandhu and Slingerland, 2000).

The treatment of cancer patients with chemotherapy is often limited by the occurrence of cancer cells resistance. Recent data suggest a possible link between cell cycle alterations and the emergence of resistance to specific anticancer agents, although the molecular mechanisms underlying such correlation still need to be elucidated (Hochhauser *et al*, 1996; Eymin *et al*, 1999).

Doxorubicin is one of the most effective agents in the treatment of breast cancer, belonging to the anthracycline family of antitumour antibiotics. This drug inhibits topoisomerase II and promotes the formation of DNA double-strand breaks; moreover, it induces, as a byproduct of its enzymatic activation, the formation of reactive oxygen species (ROS), which are believed to contribute significantly to the cytotoxic activity of the compound (Doroshow, 1983). Oxygen-derived reactive species, such as superoxide anion radical (O_2_^•−^) and hydrogen peroxide (H_2_O_2_), are important mediators of several types of cell damage, their deleterious action being physiologically counteracted by antioxidant cell defences, including vitamins, glutathione and antioxidant enzymes (Galeotti *et al*, 1990). Primary antioxidant enzymes include superoxide dismutase (SOD), catalase (CAT) and glutathione peroxidase (GPX). Sodium dismutase converts O_2_^•−^ into H_2_O_2_, which is then converted into water by CAT and GPX. There are two major forms of SOD in eukaryotic cells, a copper and zinc, containing enzyme (CuZnSOD), localised in the nucleus and cytoplasm, and a manganese-containing enzyme (MnSOD) present in the mitochondria (Galeotti *et al*, 1990). We and others previously reported that increased expression of MnSOD is associated with increased resistance to different kinds of oxidative stress. This is in particular true for cells exposed to redox-active pesticides (Paraquat) and, interestingly, to doxorubicin (Pani *et al*, 2000). These observations strongly suggest that oxidants have a major role in the cytotoxic effect of this compound, and, by extension, that antioxidant defences may contribute to cancer cell resistance to doxorubicin and other pro-oxidant drugs *in vivo*.

In the present study, we investigated the relations between the expression of cyclin E and cell response to anticancer drugs and found that increased cyclin E expression is associated with resistance to doxorubicin. We also found that this effect is associated with a reduced endogenous level of ROS and an increased expression of MnSOD. The implications of these findings are discussed.

## MATERIALS AND METHODS

### Cell culture

The Rat-1 diploid immortalised rat fibroblasts were grown and maintained in Eagle's minimum essential medium (EMEM) (Gibco, Merelbeke, Belgium) supplemented with 10% heat-inactivated FBS. Doubling times were calculated from the initial exponential phase of the growth curves. Briefly, cells were plated at a density of 1 × 10^4^ cells per 35 mm diameter well, in triplicate, and the number of cells per well was determined every day using a cell counter.

### Chemicals

Purified doxorubicin, etoposide (VP-16), methotrexate, vincristine, paclitaxel and Paraquat-dichloride were purchased from Sigma (St Louis, MO, USA). Stock solution (10 mg/ml) was prepared and storaged according with the instructions of the supplier.

### Construction of retrovirus vectors and viral transduction

The construction of the cyclin E retroviral expression plasmid PMV12-cycE and the method used for retrovirus packaging and transduction have been previously described (Sgambato *et al*, 1996, 1997). Briefly, the full-length cyclin E cDNA was subcloned into the retroviral vector PMV12 in the sense orientation (Cacace *et al*, 1993). To prepare infectious retrovirus particles, the PMV12-cycE plasmid or the control vector PMV12pl was transfected into the Ψ2 ecotropic retrovirus packaging cell lines. The transfected cells were selected by growth in hygromycin and the cell-free media, containing defective recombinant viruses, were harvested, filtered and used for the infection. Following selection for cells resistant to hygromycin, several pools of thousands of resistant colonies were obtained and used for further analysis.

### Cytotoxicity assay

Cells were plated in triplicate in 24-well plates at a density of 2 × 10^4^ cells per well and allowed to adhere 24–36 h before drug treatment. They were then rinsed and grown in medium supplemented with increasing concentrations (from 0 to 10 mM) of each drug. After 48 h, the medium was removed and cultures were incubated with medium containing 1 mg ml^−1^ MTT (3-[4,5-dimethylthiazol-2-yl]-2,5-diphenyltetrazolium bromide; Sigma) for 2 h at 37°C. The medium was then discarded and 500 *μ*l acid-isopropanol (0.04 N HCL in isopropanol) was added to each well to stop the cleavage of the tetrazolium ring by dehydrogenase enzymes that convert MTT to an insoluble purple formazan in living cells. Plates were then kept in agitation at room temperature for about 15–20 min and the level of the coloured formazan derivative was determined on a multiscan reader at a wavelength of 540 nm (reference wavelength 630 nm). Clonogenic assay was performed by seeding 500 and 1000 cells per 10 cm dish in complete medium. After overnight incubation, when cells were attached, but had not yet divided, medium was removed and, after washing with PBS, doxorubicin was added for 24 or 48 h. Afterwards, medium was changed and cultures were refed with fresh medium every 3–4 days for about 2 weeks. The cells were then fixed and stained with Giemsa and the number of grossly visible colonies was counted.

### Western blot analysis and MnSOD activity

Exponentially growing cultures of each cell lines were collected by cell scraping and cell pellets were added to 3–5 volumes of sonication buffer containing proteases and phosphatase inhibitors and sonicated at 4°C, as previously described (Sgambato *et al*, 1997; Pani *et al*, 2000). Homogenates were incubated in ice for 30 min and then centrifuged at 14 000 rpm for 15 min at 4°C. The supernatants were assayed for protein content and 50 *μ*g of protein from each sample was separated by SDS–PAGE and transferred to immobilon-P membranes. Immunodetection was performed using the enhanced chemiluminescence kit for Western blotting detection (Amersham). Assays for cyclin E-associated histone H1 kinase activity were performed as previously described (Sgambato *et al*, 1997). The polyclonal antibody to cyclin E was obtained from Upstate Biotechnology (Lake Placid, NY, USA). Anti-MnSOD and anti-CuZnSOD antibodies were from Calbiochem (Merck Eurolab GmbH, Germany). Bands were analysed on the image analysis system Gel Doc 200 System (Biorad Laboratories S.r.l., Milan, Italy) and quantitated using the Quantity One Quantitaton Software (Biorad). Manganese superoxide dismutase activity was evaluated by ‘in gel’ SOD assay on 100 *μ*g of total protein lysates, as previously described (Beauchamp and Fridovich, 1971; Pani *et al*, 2000).

### Determination of DNA content by FACS analysis

Cells were plated in duplicate in 6-cm dishes at a density of 5 × 10^5^ cells per dish and incubated 48 h. They were then trypsinised, collected and washed twice with PBS. Cell pellets were resuspended in 1 ml PBS and fixed in 5 ml of 70% ethanol. For the analysis, cells were collected by centrifugation and the pellets were resuspended in 0.2 mg ml^−1^ of propidium iodide in HBSS containing 0.6% Nonidet P-40 and RNase (50 *μ*g ml^−1^). After incubation in the dark at room temperature for 30–60 min, the cell suspension was filtered and analysed for DNA content on a Coulter EPICS 753 flow cytometer. The per cent of cells in different phases of the cell cycle was determined using the Multycicle software version 2.53.

### Measurement of ROS production

Intracellular ROS production was measured in cyclin E-overexpressing and in control cells using the oxygen radical-sensitive probe dichlorodihydrofluorescein diacetate (DCF) (Molecular Probes, Inc., Eugene, OR, USA) as described (Sgambato *et al*, 2001). Fluorescent units were measured in each well at 15-min interval for 45 min following incubation with DCF (10 *μ*M), using a Cytofluor™ 2300/2350 Fluorescence Measurement System (Millipore Corp., USA) with an excitation wavelength of 485 nm and an emission wavelength of 530 nm.

Intracellular ROS concentration was also assessed by flow cytometry on cells loaded with DCF. The dye (10 *μ*g ml^−1^) was added to cell culture 30 min before analysis. Cells were then trypsinised and green fluorescence was analysed using a Coulter Epics flow cytometer equipped with a 480 nm emission laser.

### Statistical analysis

Mean values were compared using the Wilcoxon's test. Calculations were performed using the STATA 6.0 statistical software package (Stata Corporation, College Station, TX, USA) and the results were considered statistically significant when the *P*-value was ⩽0.05.

## RESULTS

### Increased expression of cyclin E stimulates the growth of rat fibroblasts

To obtain cyclin E-overexpressing derivatives of Rat-1 fibroblasts, cells were transduced with a human cyclin E cDNA expressed from a retroviral promoter, as previously described. After selection, a pool of thousands of resistant colonies was obtained both from the cultures infected with the cyclin E cDNA construct (Rat-E) and the cultures infected with the empty vector (Rat-V, vector control cells), respectively. Pools of transfected cells were used for these studies, rather than single clones, to eliminate the possibility that our results were due to clonal heterogeneity commonly observed in cultured cells.

**Figure 1:**
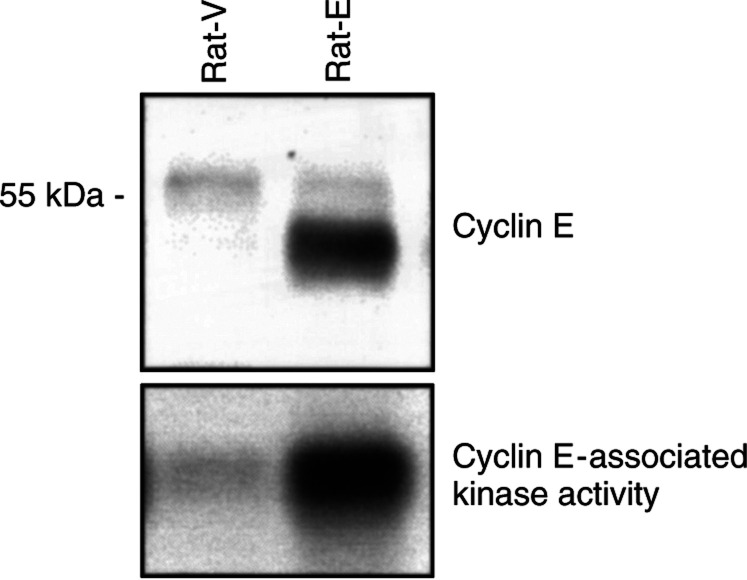
Levels of expression of cyclin E and cyclin E-associated kinase activity in Rat-V and Rat-E cells. Top panel, cell extracts from exponentially growing cultures of both cell lines were analysed for the expression of cyclin E using a specific anticyclin E antibody. The major endogenous cyclin E band was about 55 kDa in the rat fibroblasts. A strong band of about 50 kDa corresponding to the exogenous cyclin E was detected in the overexpressing pool. The size difference between the human and rodent forms was a convenient property for distinguishing the endogenous and exogenous cyclin E. Bottom panel, cell extracts from both cell lines were assayed for the cyclin E-associated histone H1 kinase activity.

Expression of the exogenous cyclin E gene was verified by Western blot analysis with a specific antibody to cyclin E. The major endogenous cyclin E band was about 55 kDa in the rat fibroblasts. A strong band of about 50 kDa corresponding to the exogenous cyclin E was also detected in the overexpressing pool but not in the vector control cells (Figure 1

). The size difference between the human and rodent forms was a convenient property for distinguishing the endogenous and exogenous cyclin E. To determine the effects of cyclin E overexpression on CDK activity, lysates from both pools were assayed for cyclin E-associated kinase activity, which resulted in significantly higher levels in Rat-E cells compared with vector control cells (Figure 1). No differences were observed in the doubling time of cyclin E-overexpressing cells compared with Rat-V control and parental cells (about 18 h, data not shown). However, as expected, the cyclin E-overexpressing derivatives of Rat-1 fibroblasts displayed a reduction in the percentage of cells in the G1 phase (about 36 *vs* 62%) and an increase in the percentage of cells in the S phase (about 47 *vs* 22%), when compared to the vector control cells (Table 1

**Table 1:** Cell cycle distribution in cyclin E-overexpressing fibroblasts compared with vector control cells

	**G0/G1**		**S**		**G2/M**	
**Cell lines**	**%cells^b^**	**Time(h)^a^**	**%cells^b^**	**Time(h)^a^**	**%cells^b^**	**Time(h)^a^**
Rat-1	65.4	11.8	18.2	3.3	16.4	2.9
Rat-V	62.3	11.2	21.8	3.9	15.9	2.9
Rat-E	36.4	6.6	47.3	8.5	16.3	2.9

Exponentially growing cultures of the indicated cell lines were analysed by flow cytometry.

a The values indicate the length of each phase of the cell cycle based on the doubling time of the indicated cell line.

b The values represent the percentage of the total cell population in each phase of the cell cycle. The data reported are the results of a typical experiment. Similar results were obtained in replicate experiments.

). As shown, no significant differences in cell cycle distribution were observed between Rat-V and Rat-1 parental cells. Based on the length of the exponential doubling time, we used the flow cytometry data to calculate the approximate length in hours of each of the phases of the cell cycle in the two cell lines. As shown in Table 1, the cyclin E-overexpressing cells displayed a shortening of the G1 phase associated with lengthening of the S phase of the cell cycle.

### Cyclin E overexpression is associated with an increased resistance to doxorubicin but not to other antineoplastic agents

**Figure 2:**
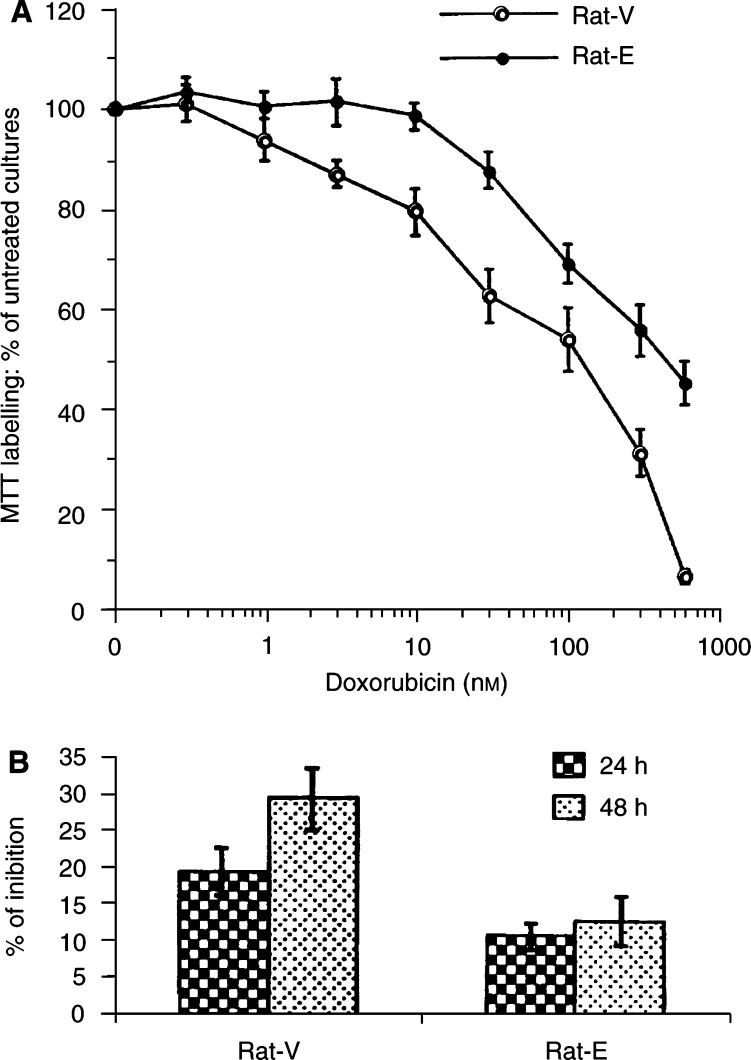
Increased resistance of cyclin E-overexpressing cells to doxorubicin-induced inhibition of cell growth. (**A**) Cells were exposed to the indicated concentration of doxorubicin and cell numbers were estimated by the MTT test after 48 h and plotted as a function of controls without drugs. (**B**) Chemosensitivity to doxorubicin was assessed by clonogenic assay. Cells were exposed to doxorubicin for 24 and 48 h and results were expressed as percentage of inhibition in colony formation in drug-treated compared with untreated cells. See Materials and Methods for details.

To evaluate whether the increase in cyclin E expression affected sensitivity of cells to anticancer drugs, exponentially growing cultures of both vector control and cyclin E-overexpressing derivatives were exposed to increasing concentration of drugs (methotrexate, etoposide, paclitaxel, vincristine and doxorubicin) for 48 h and the concentration inhibiting cell growth by 50% (IC_50_) was determined. Cytotoxicity assays were carried out by use of the MTT test. A dose-dependent decrease in cell number was observed with all the five drugs. As shown in Figure 2A

, cyclin E-overexpressing fibroblasts were more resistant to doxorubicin compared with vector control cells (the IC_50_ value was 1.9-fold higher) (*P*<0.0001). The effect was specific since no significant difference in growth inhibition was evident between Rat-V and Rat-E for methotrexate, etoposide, paclitaxel and vincristine even when drug treatment was prolonged up to 72 h (Table 2

**Table 2:** Growth inhibition by antineoplastic drugs in cyclin E-overexpressing fibroblasts compared with vector control cells

	**IC_50_**	
	**Rat-V**	**Rat-E**
Methotrexate	346.3±31.2	296.6±43.2
Doxorubicin	240.2±19.5	463.1±36.2*
Paclitaxel	60.6±12.5	71.9±25.6
Vincristine	326.4±12.7	327.8±14.8
VP-16	31.7±6.3	25.8±5.6

a The values represent the means±s.d. of four different experiments performed in triplicate. The IC_50_ is the concentrations of drug inhibiting cell growth by 50% in the MTT assay at 48 h after drug treatment. Comparable results were obtained at 72 h treatment (data not shown). Concentrations are expressed as *μ*M for VP-16 and as nM for all the other drugs.

* Significant at *P*<0.0001.

and data not shown). The increased resistance of cyclin E-overexpressing derivatives was also evident after 72 h incubation with doxorubicin (IC_50_=122.1±9.7 and 193.3±20.1, respectively) (*P*<0.0001) (data not shown). Confirmatory results were obtained when Rat-E and Rat-V cells were incubated in media at different concentration of doxorubicin and cell growth was analysed up to 72 h by cell counting (data not shown). Chemosensitivity to doxorubicin was also assessed by clonogenic assay, which is a better predictor of *in vivo* sensitivity compared with MTT test. Different concentrations of drug were tested for 24 and 48 h treatment and inhibition in colony formation was always higher for control compared with cyclin E-overexpressing cells (data not shown). At a concentration of 25 nM doxorubicin, colony formation was reduced about 20 (19.3±4) and 11 (10.8±2)% after 24 h and 30 (29.5±5) and 13 (12.7±4)% after 48 h treatment for Rat-V and Rat-E cells, respectively, compared with untreated cells (Figure 2B). We analysed the effects of drug treatment on cell cycle distribution and found that treatment of cells with doxorubicin (IC_50_) for 24 h mainly caused an accumulation of cells in the G1 phase of the cell cycle in both the Rat-V (G0/G1=73.4; S=14.7; G2/M=11.9) and Rat-E cells (G0/G1=60.4; S=26.8; G2/M=12.8), compared with untreated cells. We also looked for apoptosis by FACS and TUNEL analysis in doxorubicin-treated cells, but were unable to detect apoptotic cells in both Rat-V and Rat-E cells up to 48 h treatment with the IC_50_. We cannot exclude that apoptosis might be apparent at later time points or with higher drug concentration (data not shown).

### Increased expression of cyclin E is associated with a reduction in ROS

Doxorubicin belongs to anthracycline antibiotics that have been shown to induce cytotoxicity by generating ROS and whose activity is influenced by the redox state of cells (Doroshow, 1983; Sinha *et al*, 1987; Friesen *et al*, 1999).

**Figure 3:**
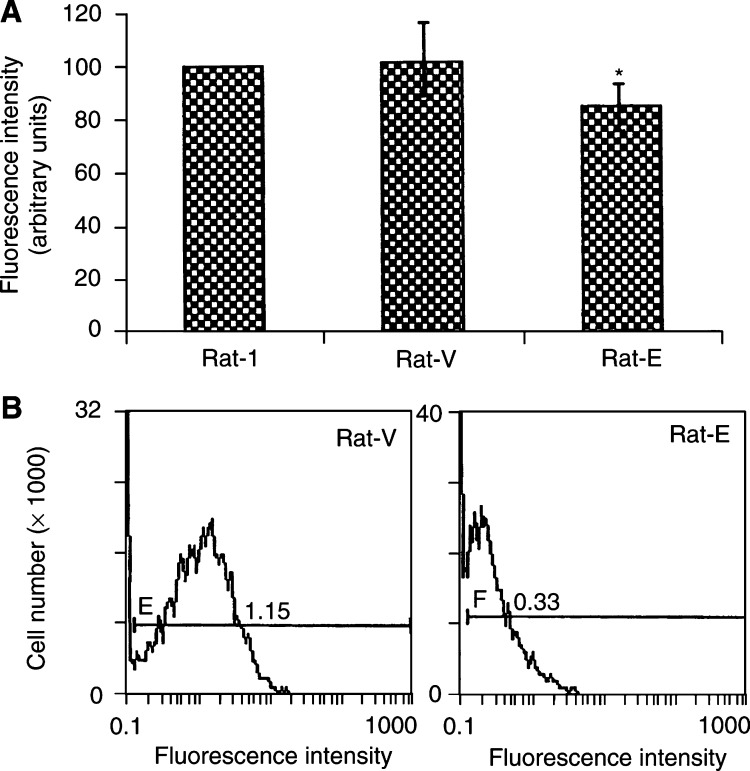
Reduced concentration of intracellular ROS in cyclin E-overexpressing cells. (**A**) dichlorodihydrofluorescein diacetate fluorescence was measured after 45 min incubation with DCF (10 *μ*M). The values represent the means±s.d. of four different experiments performed in triplicate and are shown as per cent of Rat-1 control cells. Similar values were obtained taking into account the readings after 15 and 30 min incubation with DCF (data not shown). ^*^Significant at *P*=0.001 for Rat-E *vs* Rat-V. (**B**) Intracellular ROS concentration was assessed by flow cytometry on cells loaded with DCF. The dye (10 *μ*g ml^−1^) was added to cell culture 30 min before analysis. Cells were then detached from the substrate and green fluorescence was analysed using a Coulter Epics flow cytometer equipped with a 480 nm emission laser. Numbers beside the histograms indicate mean cell fluorescence and are the averages of three independent experiments.

To evaluate whether cyclin E overexpression was associated with changes in the redox state of rat fibroblasts, we analysed the oxidative state of both cyclin E-overexpressing and control cells using the fluorescent probe DCF, which is a sensitive fluorimetric probe of the production of oxidative stress in living cells (Zhu *et al*, 1994). We found that the basal level of endogenous ROS was significantly lower (about 16%) (*P*=0.001) in cyclin E-overexpressing cells compared with vector control and parental cells (Figure 3A

). The reduced level of ROS was also confirmed using a fluorescence microscope (data not shown) and by flow cytometric (FACS) analysis of cells after incubation with DCF for 45 min (Figure 3B).

### Reduced induction of free radical production by doxorubicin in cyclin E-overexpressing cells

Since generation of ROS has been suggested as a main mechanism of anthracycline cytotoxicity (Doroshow, 1983; Sinha *et al*, 1987), we asked whether doxorubicin-induced increase in ROS level was also reduced in cyclin E-overexpressing cells compared with vector control cells. Although both cell lines displayed a marked increase in ROS levels following doxorubicin treatment, it was about 1.9-fold for Rat-V and 1.7-fold for Rat-E cells with an increase of 107 and 67 fluorescence units, respectively. It is of interest, however, that the absolute values remained constantly lower in Rat-E compared to Rat-V cells after drug treatment (*P*=0.03 after 4 h by Wilcoxon test) (Table 3

**Table 3:** Increase in the level of endogenous ROS following doxorubicin treatment in cyclin E-overexpressing fibroblasts compared with vector control cells

	**DCF fluorescence (arbitrary units)**	
	**Rat-V**	**Rat-E**
Basal	100	83±3
Doxo 15 min	110±9	93±13*
Doxo 4 h	203±26	160±18**
H_2_O_2_	250±29	170±16**

a Dichlorodihydrofluorescein diacetate fluorescence was measured after 45 min incubation with DCF (10 *μ*M). Cells were exposed to doxorubicin (Doxo, IC_50_) or to H_2_O_2_ (100 *μ*M for 15 min) before the analysis. The values are given as per cent of Rat-V control cells and represent the means±s.d. of four different experiments performed in triplicate. Similar values were obtained taking into account the readings after 15 and 30 min incubation with DCF (data not shown).

* *P*=0.07.

** Significant at *P*=0.03.

). This effect was not specific for doxorubicin. In fact, exposure to H_2_O_2_ (100 *μ*M for 15 min) also caused a smaller increase in ROS level in cyclin E-overexpressing cells compared to vector control cells (*P*=0.003) (Table 3).

### Cyclin E overexpression is associated with an increased expression of MnSOD

**Figure 4:**
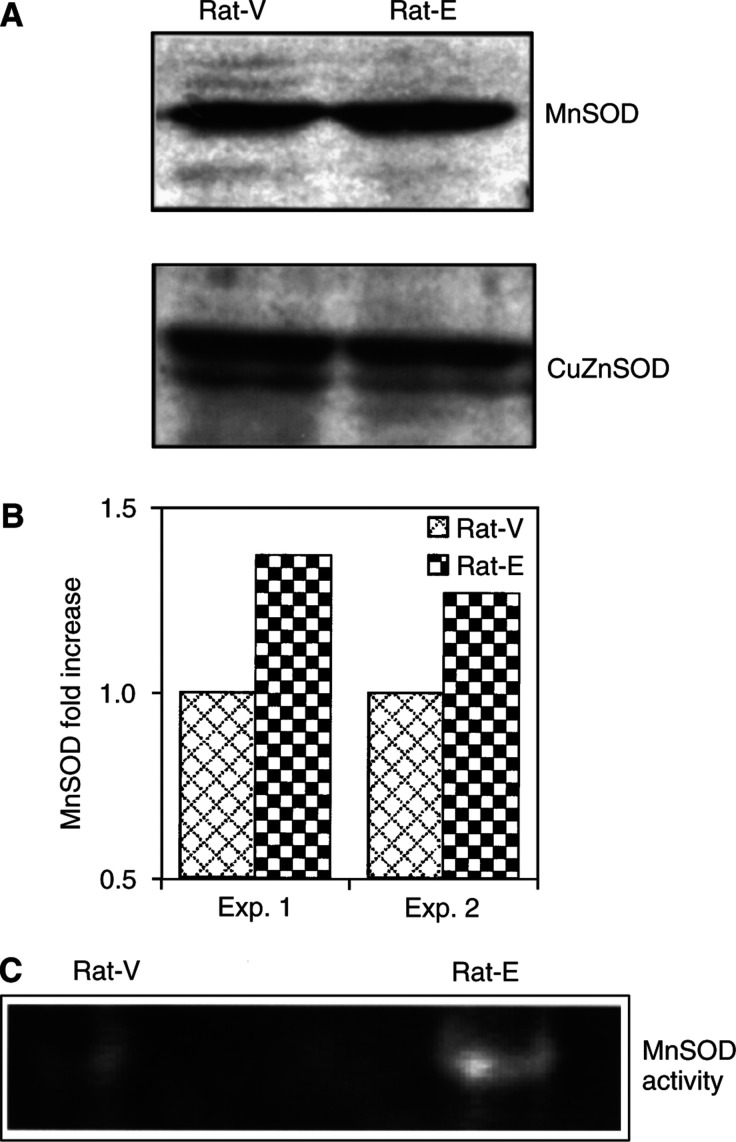
Increased expression and activity of MnSOD in cyclin E-overexpressing cells. (**A**) Cell extracts from exponentially growing cultures of both the cell lines were analysed for the expression of MnSOD and CuZnSOD by Western blot analysis. (**B**) Densitometric analysis of MnSOD bands. The values shown are the ratios of MnSOD to *β*-actin obtained in two independent experiments. (**C**) Manganese superoxide dismutase activity was assessed by gel SOD assay, as described by Beauchamp and Fridovich (1971).

Manganese superoxide dismutase is the principal scavenger for superoxide in mitochondria (Fridovich, 1998). Since increased expression of MnSOD has been associated with improved survival of cells to doxorubicin (Hirose *et al*, 1993), we aimed to verify whether changes in the expression level of this protein played a role in the increased resistance of cyclin E-overexpressing fibroblast to doxorubicin. The expression of MnSOD was evaluated by Western blot analysis in Rat-E and Rat-V cells. As shown in Figure 4A and B

, cyclin E-overexpressing derivatives expressed an increased level (about 1.3-fold) of expression of MnSOD protein compared with Rat-V vector control cells. Accordingly, MnSOD activity was also significantly increased (about 40%) in Rat-E cells, compared with control cells, as assessed by gel SOD assay (Figure 4C). No differences were observed in the expression of CuZnSOD (Figure 4A).

## DISCUSSION

In this study, we demonstrated that cyclin E overexpression, which is a frequent event in a variety of human malignancies, is associated with an increased resistance to doxorubicin-induced cell death. Cyclin E is a major regulator of G1 to S transition in eukaryotic cells (Koff *et al*, 1991; Ohtsubo *et al*, 1995) and its increased expression has been reported in a variety of human malignancies (Donnellan and Chetty, 1999; Sandhu and Slingerland, 2000). In this study, we analysed the sensitivity to doxorubicin and to other anticancer drugs of derivatives of Rat-1 fibroblasts that stably overexpress cyclin E and demonstrated that changes in the expression levels of cyclin E can affect cell sensitivity to doxorubicin.

Doxorubicin is a chemotherapeutic agent widely used in the treatment of human cancers (DeVita *et al*, 2001). It belongs to the anthracycline family of anticancer antibiotics that can inhibit topoisomerase II action resulting in double-strand DNA breaks (Kasahara *et al*, 1992). However, the generation of ROS has been suggested as the major mechanism of anthracycline cytotoxicity. The quinone, which is functionally common to the members of this family, may undergo a one-electron reduction to the corresponding semiquinone free radical by flavin-centred reductases including NADH dehydrogenase (Doroshow, 1983). In the presence of oxygen, this free radical will trigger the generation of highly ROS that can induce lipid peroxidation and DNA damage. The enzymes catalase, SOD and the glutathione system play a key role in the cellular defence against ROS damage by detoxifying some of these reactive molecules before they react with vulnerable cellular targets (Fridovich, 1998). ROS generation as a result of doxorubicin treatment and prevention of its cytotoxicity by oxygen radical scavengers was directly demonstrated in MCF-7 human breast cancer cells (Doroshow, 1986; Bustamante *et al*, 1990).

The present study demonstrates that increased expression of cyclin E in rat fibroblasts is associated with an increased resistance to doxorubicin, but not to other antineoplastic agents, such as methotrexate, etoposide, vincristine and cysplatin. We also demonstrated that this effect likely relates to a reduced basal level of endogenous ROS and to their reduced increase following doxorubicin treatment in cyclin E-overexpressing derivatives compared with vector control cells (Figure 3 and Table 3). Reactive oxygen species are, in fact, general mediators of cell damage (Kehrer, 1993) and their generation is a prerequisite for the triggering of cell death by several stimuli, including growth factor withdrawal (Palazzotti *et al*, 1999) and activation of the tumour suppressor protein p53 (Polyak *et al*, 1997). Since both endogenous levels of ROS and intracellular oxidations induced by exogenous oxidants (H_2_O_2_ and doxorubicin) are diminished in Rat-E cells, these differences likely reflect an increased capacity of oxidant scavenging in cells overexpressing cyclin E. This hypothesis is supported by the observation that the protein expression and enzyme activity of the mitochondrial scavenger MnSOD, one of the major enzymes involved in the cellular defence against ROS, are upregulated (30% increase) in these cells compared with the corresponding controls (Figure 4). The observation that Rat-E did not display a reduced sensitivity to etoposide, which is a specific topoisomerase inhibitor, further supports our hypothesis that the increased resistance to doxorubicin is likely due to a reduced susceptibility of cells to the ROS-generating ability of the drug rather than to its inhibitory activity on topoisomerase II enzyme.

The mechanisms responsible for the increase of MnSOD expression in cyclin E-overexpressing cells remain unknown. However, it is of interest that some pools displayed a loss of expression of the exogenous cyclin E with prolonged serial passages that was associated with a decrease in the expression of MnSOD to the normal basal level (data not shown), thus further confirming the link between the two events. Moreover, we analysed by immunostaining the expression of the p53 protein in the Rat-V and Rat-E cells and found that both cell lines displayed a weak basal level of p53 nuclear staining that increased following doxorubicin treatment (data not shown). Thus, an involvement of the p53 protein in the MnSOD upregulation in cyclin E-overexpressing cells is unlikely. In preliminary experiments, we did not observe by Northern blot analysis any difference in the expression levels of MnSOD mRNA between cyclin E-overexpressing and vector cell lines (unpublished results). However, further studies are required to establish definitively the mechanism(s) responsible for the increase of MnSOD protein in cyclin E transfected cells, which might involve modification at the transcriptional, translational or post-translational levels.

We propose that the increased expression of MnSOD observed in Rat-E cells contributes to the resistance of these cells to doxorubicin. In fact, (a) we and others have previously reported that increased expression of MnSOD at levels comparable to those observed in Rat-E cells is associated with improved survival of tumour cells exposed to doxorubicin *in vitro* (Hirose *et al*, 1993; Pani *et al*, 2000) and an increased expression of SOD was previously reported in doxorubicin-resistant Friend leukaemic (about 24%) (Crescimanno *et al*, 1991) and breast cancer (Zyad *et al*, 1994) cell lines and in leukaemic patients (Watanabe, 1998); (b) compared with vector control cells, cyclin E-overexpressing Rat cells were also more resistant to Paraquat (IC_50_=370.9±9 *vs* 289.3±15.5 *μ*M) (data not shown), a superoxide-generating compound whose action is inhibited by SODs (Bus *et al*, 1974; Pani *et al*, 2000); thus further confirming the biological significance of the increase in MnSOD expression and activity observed in cyclin E-overexpressing cells compared with control cells. The finding that the CuZnSOD is not increased in Rat-E cells further confirms that MnSOD is selectively upregulated in cyclin E-overexpressing cells (Figure 4).

One of the major mechanisms of resistance to doxorubicin is due to the acquisition of the so-called multiple drug resistance (MDR) phenotype which, in most of the cases, depends on the expression of a transmembrane 170 kDa glycoprotein, called P-glycoprotein (Kartner *et al*, 1983). Cells that develop resistance through the MDR mechanism, however, simultaneously develop cross-resistance to several structurally unrelated natural products, including anthracyclines, vinka alkaloids, epipodophyllotoxins, taxanes and actinomycin-D (Kartner *et al*, 1983). Since the Rat-E cells only displayed an increased resistance to doxorubicin, but not to the vinca alkaloid vincristine, nor to paclitaxel, the acquisition of an MDR-phenotype in these cells is unlikely. However, while in light of the above consideration an involvement of MnSOD in Rat-E cell resistance to doxorubicin appears to be conceivable, the present results do not allow, at the moment, to exclude that other mechanisms, that is, involving ROS production and/or their detoxification or DNA repair, are also affected by cyclin E overexpression.

Although obtained in diploid immortalised rat fibroblasts, we believe that the results of the present study are instructive in revealing a potential new mechanism of resistance to doxorubicin in cancer cells. Regardless of the underlying molecular mechanisms, if it will be confirmed, the observed relation between the expression levels of cyclin E and cell sensitivity to doxorubicin might have important implications for the treatment of cancer, mainly breast cancer patients. In fact, doxorubicin is one of the most valuable anticancer drug in present day clinical use, being an integral part of the treatment of malignancies such as carcinoma of the breast, head and neck, thyroid and soft tissue sarcomas and leukaemias (DeVita *et al*, 2001). Moreover, on the other hand, increased expression of cyclin E is a common event in a variety of human malignancies (Donnellan and Chetty, 1999; Sandhu and Slingerland, 2000) and is believed to play an important role in cancer development and progression by causing cell growth deregulation and genomic instability (Bortner and Rosenberg, 1997; Spruck *et al*, 1999). Thus, it has been proposed that cyclin E may become a target for treatment of human cancers (Owa *et al*, 2001). Increased expression of cyclin E has been reported associated with poor prognosis in breast cancer patients (Nielsen *et al*, 1996; Porter *et al*, 1997). Since doxorubicin is included in most of the chemoterapeutic regimens for treatment of breast cancers, our results might also suggest that, at least in some cases, the reported relation between high cyclin E levels and poor prognosis in breast cancer patients might simply reflect the reduced responsivity of cyclin E-overexpressing tumours to doxorubicin (Keyomarsi *et al*, 1994; Nielsen *et al*, 1996). Further *in vivo* studies will be needed to address this point.

In conclusion, the results of the present study suggest that evaluation of cyclin E expression levels might help to select the most appropriate treatment for cancer patients, mainly breast cancer patients, by reserving anthracycline treatment to patients whose tumours do not overexpress this molecule. These results are in agreement with previous studies on the role of cyclin E and other cell cycle regulatory proteins (i.e. the CDK inhibitor p27^Kip1^) in determining the response of cells to cytotoxic agents and warrant further studies in this interesting area of cancer research (Croix *et al*, 1996; Smith and Seo, 2000; Yang *et al*, 2000).

Furthermore, the involvement of cyclin E and MnSOD in cell resistance to doxorubicin suggests that therapeuthical strategies (i.e. antisense or chemical inhibitors) targeting cyclin E/CDK (Sausville *et al*, 2000) or SOD activities (Huang *et al*, 2000) could also be tested as chemosensitising tools to anthracycline treatment in cyclin E-overexpressing tumours.
